# Dynamics of Germinosome Formation and FRET-Based Analysis of Interactions between GerD and Germinant Receptor Subunits in *Bacillus cereus* Spores

**DOI:** 10.3390/ijms222011230

**Published:** 2021-10-18

**Authors:** Yan Wang, Ronald M. P. Breedijk, Mark A. Hink, Lars Bults, Norbert O. E. Vischer, Peter Setlow, Stanley Brul

**Affiliations:** 1 Molecular Biology and Microbial Food Safety, Swammerdam Institute for Life Sciences, University of Amsterdam, Science Park 904, 1098 XH Amsterdam, The Netherlands; y.wang5@uva.nl (Y.W.); larsbults@hotmail.com (L.B.); norbertvischer@gmail.com (N.O.E.V.); 2 Van Leeuwenhoek Centre for Advanced Microscopy, Molecular Cytology, Swammerdam Institute for Life Sciences, University of Amsterdam, Science Park 904, 1098 XH Amsterdam, The Netherlands; R.M.P.Breedijk@uva.nl (R.M.P.B.); m.a.hink@uva.nl (M.A.H.); 3 Department of Molecular Biology and Biophysics, UConn Health, Farmington, CT 06030-3305, USA; setlow@nso2.uchc.edu

**Keywords:** *Bacillus cereus*, spore, sporulation, germination proteins, FRET

## Abstract

Spores of the bacterium *Bacillus cereus* can cause disease in humans due to contamination of raw materials for food manufacturing. These dormant, resistant spores can survive for years in the environment, but can germinate and grow when their surroundings become suitable, and spore germination proteins play an important role in the decision to germinate. Since germinated spores have lost dormant spores’ extreme resistance, knowledge about the formation and function of germination proteins could be useful in suggesting new preservation strategies to control *B. cereus* spores. In this study, we confirmed that the GerR germinant receptor’s (GR) A, B, and C subunits and GerD co-localize in *B. cereus* spore inner membrane (IM) foci termed germinosomes. The interaction between these proteins was examined by using fusions to the fluorescent reporter proteins SGFP2 and mScarlet-I and Förster Resonance Energy Transfer (FRET). This work found that the FRET efficiency was 6% between GerR(A-C-B)–SGFP2 and GerD–mScarlet-I, but there was no FRET between GerD–mScarlet-I and either GerRA–SGFP2 or GerRC–SGFP2. These results and that GerD does not interact with a GR C-subunit in vitro suggest that, in the germinosome, GerD interacts primarily with the GR B subunit. The dynamics of formation of germinosomes with GerR(A-C-B)–SGFP2 and GerD–mScarlet-I was also followed during sporulation. Our results showed heterogeneity in the formation of FRET positive foci of GerR(A-C-B)–SGFP2 and GerD–mScarlet-I; and while some foci formed at the same time, the formation of foci in the FRET channel could be significantly delayed. The latter finding suggests that either the GerR GR can at least transiently form IM foci in the absence of GerD, or that, while GerD is essential for GerR foci formation, the time to attain the final germinosome structure with close contacts between GerD and GerR can be heterogeneous.

## 1. Introduction

*Bacillus cereus*, a member of the bacterial *B. cereus sensu lato* group, is a toxin-producing spore former which is widely distributed in the environment and commonly present in many raw foodstuffs, including rice, eggs, and milk. These resistant spores can survive in improperly processed foods and then germinate, grow, and produce toxins which can cause disease in humans [1,2,3,4]. In general, *B. cereus* spores are formed upon limitation of nutrients in the environment, just as in the model spore-former *Bacillus subtilis* [5,6,7]. Compared with vegetative cells, *Bacillus* spores have a very different morphology, with many spore-specific layers. The innermost layer is the core containing chromosomal DNA saturated by protective small acid-soluble spore proteins (SASPs) and surrounded by the inner membrane (IM), which contains a number of spore-specific proteins. Then there are two peptidoglycan (PG) layers, first the germ cell wall, which is identical to vegetative cell PG, and then the much larger cortex PG layer, which has some spore-specific features. Next, there is the outer membrane, and then the various layers of the coat made up of spore-specific proteins [8]. Notably, *B. cereus* spores are also surrounded by a balloon-like exosporium, which is composed largely of spore-specific lipoproteins and glycoproteins; this exosporium layer is found in spores of all members of the *B. cereus sensu lato* group, including *Bacillus anthracis* [9,10]. The various spore-specific structures and components all contribute to spore dormancy, as well as spores’ high resistance to agents, such as wet and dry heat, desiccation, UV and γ-radiation, and multiple types of toxic chemicals [11].

During spore formation, many proteins involved in spore germination, including a group of nutrient germinant receptors (GRs), are assembled and localized in the IM of the nascent spore or forespore [12,13,14,15,16]. The GRs allow dormant spores to continually monitor nutrients in their surroundings, as GRs respond to low-molecular-weight nutrients, commonly specific amino acids, sugars, and purine nucleosides to trigger germination. Once germination has been initiated, spores rapidly release their huge depot of dipicolinic acid in a 1:1 complex with Ca^2+^ (CaDPA; ~25% of spore core dry wt) and then degrade cortex PG, take up core water, resume metabolism, and finally outgrow into vegetative cells [17]. In addition to GRs, there are some other key IM germination proteins: GerD, which forms a complex with GRs [18,19], and SpoVA channel proteins, which are important for CaDPA uptake in sporulation and its release during germination [20,21].

*B. subtilis* spores contain three functional GRs, namely GerA, GerB, and GerK, with GerA responsible for germination with L-alanine or L-valine [22]. Seven GRs have been identified in *B. cereus* spores: GerR, GerK, GerG, GerL, GerQ, GerI, and GerS, with GerR triggering germination with L-alanine [23]. GRs from all species contain A, B, and C subunits; the A and B subunits are integral IM proteins, although the A subunit also has a large hydrophilic domain, and the hydrophilic C subunit is held in the spore IM by a diacylglyceryl anchor [24,25].

Previous work showed that GRs are present in the *B. subtilis* spore IM in one or two foci, termed germinosomes, which also contain GerD. Most importantly, the GR GerKB fused to mCherry and GerD fused to GFP co-localized in the IM foci in *B. subtilis* spores, and the clustering of GRs required all three GR subunits, as well as GerD [19,26]. However, interactions between GRs and GerD are poorly understood in *B. subtilis* spores, and there is no information about such interactions in *B. cereus* spores, although GR proteins and GerD also co-localize in a few foci in these spores [15].

Förster resonance energy transfer (FRET) is often used to analyze the interaction of two proteins based on the transfer of fluorescence energy between two fluorescent proteins. In the current work, we chose to use variants of green and red fluorescent proteins as FRET pairs. The reasons for the choice of the specific proteins are that previous studies showed that strongly enhanced green fluorescent protein (SGFP2) has increased brightness, maturation efficiency and photostability [27], and the red fluorescent protein mScarlet-I also exhibits high brightness and greater maturation efficiency than the original mScarlet [28]. Consequently, we aimed to localize germination proteins in *B. cereus* forespores’ IM and analyze the interaction between GerR GR subunits and GerD by expressing fusions of GR subunits with SGFP2 and GerD with mScarlet-I under the control of the native promoters on plasmids and using filter FRET. The dynamics of germinosome foci formation by GerR(A-C-B)–SGFP2 and GerD–mScarlet-I during sporulation were also recorded by time-lapse microscopy. Our results indicated that fusions of all GerR subunits and GerD are localized in the IM of *B. cereus* spores based on the co-localization of protein fusions and the FM 4-64 membrane dye. Importantly, the fusions to the GerRB protein and GerD exhibited FRET interaction, which was not seen between the GerD fusion and either GerRA or GerRC fusions, suggesting that it is the GR B subunit that interacts with GerD. Notably, the appearance of the interaction signal from the FRET channel during forespore development was sometimes significantly slower than the formation of GerR(A-C-B) and GerD foci, suggesting that there is significant kinetic heterogeneity in the formation of the final germinosome structure.

## 2. Results

### 2.1. B. cereus Expressing SGFP2 or/and mScarlet-I Fusion Proteins

**Figure 1:**
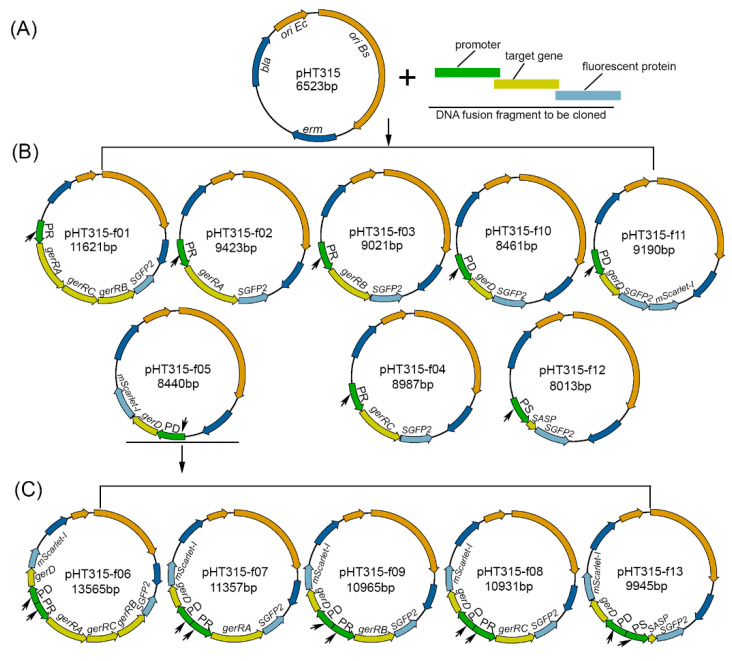
Maps of recombinant plasmids used in this study. The empty vector pHT315 of layer (**A**) was used as the backbone for construction of plasmids in layer (**B**). Plasmid pHT315-f05 of layer B was used as the backbone to construct plasmids in layer (**C**). The black arrows indicate the locations of promoter sequences for fusion genes. The recombinant genes in each of these plasmids are listed in Table 1.

The GerR subunits A, B, and C and GerD were visualized in dormant *B. cereus* spores, using reporter proteins SGFP2 and mScarlet-I, as described in previous work [15]. The successful use of fluorescent reporter proteins to demonstrate germinosomes in *B. cereus* [15] suggests them to be functional. A major aim of the new studies was to interrogate the interactions between each GerR GR subunit with the so-called germinosome scaffold protein GerD, using recombinant plasmids containing the C-terminus of the gene of interest fused to SGFP2 or mScarlet-I and controlled by the native promoter of the gene of interest (Figure 1 and Table 1).

**Figure 2:**
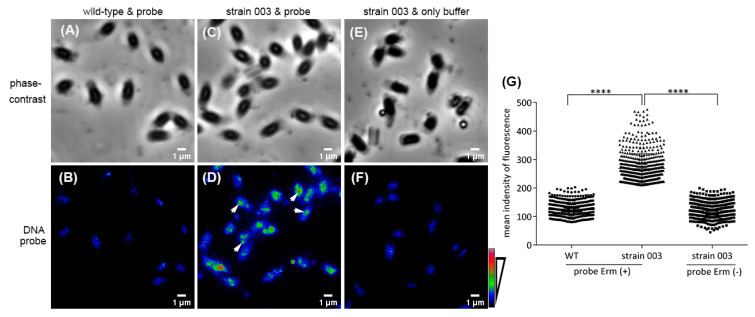
FISH detection of plasmid DNA in spores of *B. cereus* strain 003. Spores of *B. cereus* wild-type and strain 003 were analyzed by FISH, and phase-contrast (panels (**A**,**C**,**E**) and probed images (panels (**B**,**D**,**F**) were obtained. Spores of strain 003 hybridized, using buffer, without the DNA probe, as a negative control are shown in panels (**E**,**F**). The mean fluorescence intensity of 670 individual spores of each strain is shown in panel (**G**). The images of DNA probed samples were given a false color of rainbow RGB (panels (**B**,**D**,**F**); and see scale bar on lower right). The arrows in panel (**D**) indicate some locations of plasmid hybridized with the *erm* probe. Note that the spores appear dark in the phase-contrast images because of the processing for the FISH detection. ****, *p* < 0.0001.

To confirm that at least one plasmid copy was present in spores of *B. cereus* strain 003, fluorescence in situ hybridization (FISH) analysis was performed by using a DNA probe from this strain’s plasmid’s erythromycin resistance gene (*erm*) (Figure 2). The results showed that almost all spores of strain 003 gave bright spots, using the DNA probe (Figure 2E). However, due to the small size of *B. cereus* spores (1.2~2 µm) [29], the number of a single spots in each spore could not be reliably counted. Nevertheless, the analysis of the fluorescence intensity of 670 spores of strain 003 and the plasmid-free wild-type spores probed with the *erm* gene fragment and strain 003 spores probed without the *erm* fragment showed that the *erm* labeling intensities in spores of strain 003 were significantly higher than the fluorescence in either wild-type spores or *erm*-free probed strain 003 spores (Figure 2G). Spores of strain 003 hybridized with the *erm* probe also had a large range of intensity distribution in single spores (Figure 2G), suggesting that there is significant variability in plasmid copy numbers in these spores. The results of this study were thus in line with our previous work [15] and indicated that there is at least one plasmid copy in almost all spores of *B. cereus* strain 003, and presumably also in strains transformed with the other, similar plasmids.

**Table 1:** Strains and plasmids used in this study.

Strains	Plasmid Present (+)	Description of Inserted Genes	Sources
*Escherichia coli* strains			
DH5α	wild type		Lab stock
DH5α 01	pSGFP2-C1 Kan^r^	*SGFP2*	Lab stock
DH5α 02	pEB2-mScarlet-I Kan^r^	*mScarlet-I*	Lab stock
*Bacillus cereus* strains			
*B. cereus* ATCC 14579	wild type	No	Lab stock
strain 006	+pHT315-f01 Ery^r^	PR-*gerR(A-C-B)–SGFP2*	This study
strain 008	+pHT315-f02 Ery^r^	PR-*gerRA–SGFP2*	This study
strain 003	+pHT315-f03 Ery^r^	PR-*gerRB–SGFP2*	This study
strain 009	+pHT315-f04 Ery^r^	PR-*gerRC–SGFP2*	This study
strain 007	+pHT315-f05 Ery^r^	PD-*gerD*–*mScarlet-I*	This study
strain 010	+pHT315-f10 Ery^r^	PD-*gerD*–SGFP2	This study
strain 012	+pHT315-f12 Ery^r^	PS-*SASP*–*SGFP2*	This study
strain F06	+pHT315-f06 Ery^r^	PR-*gerR*(A-C-B)–SGFP2 and PD-*gerD–mScarlet-I*	This study
strain F07	+pHT315-f07 Ery^r^	PR-*gerRA–SGFP2* and PD-*gerD*–*mScarlet-I*	This study
strain F08	+pHT315-f08 Ery^r^	PR-*gerRC–SGFP2* and PD-*gerD*–*mScarlet-I*	This study
strain F09	+pHT315-f09 Ery^r^	PR-*gerRB–SGFP2* and PD-*gerD*–*mScarlet-I*	This study
strain F11	+pHT315-f11 Ery^r^	PD-*gerD*–SGFP2–*mScarlet-I*	This study
strain F13	+pHT315-f13 Ery^r^	PS-*SASP*–*SGFP2* and PD-*gerD*–*mScarlet-I*	This study

Abbreviations: PR, promoter of *gerR* operon; PD, promoter of *gerD*; PS, promoter of the SASP gene; Ery^r^, resistant to erythromycin; Kan^r^, gene for resistant to kanamycin.

### 2.2. Localization of Putative Germinosomes in Developing B. cereus Forespores

**Figure 3:**
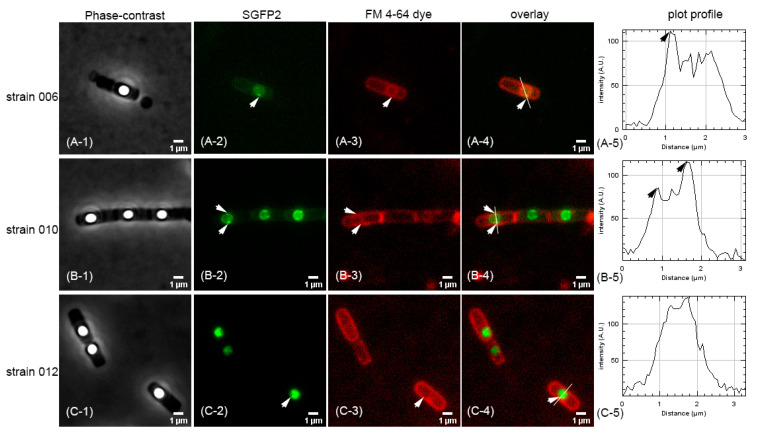
Wide-field microscopy imaging of *B. cereus* strains 006, 010, and 012 forespores stained with FM 4-64. Panels ((**A-1**),(**B-1**),(**C-1**))—phase-contrast images. Panels ((**A-2**),(**B-2**),(**C-2**))—foci of fusion proteins GerR(A-C-B)-SGFP2, GerD-SGFP2 (to allow visualization of this fusion in the presence of FM 4-64) and SASP-SGFP2 in developing forespores. Panels ((**A-3**),(**B-3**),(**C-3**))—FM 4-64 staining of the IM of developing forespores. Panels ((**A-4**),(**B-4**),(**C-4**))—merged foci in the channel between SGFP2 and FM 4-64 images. Panels ((**A-5**),(**B-5**),(**C-5**))—plot profiles of the lines from merged images using ImageJ. The arrows in the various panels indicate positions of single foci in forespores. These samples were prepared and imaged as described in Methods.

**Figure 4:**
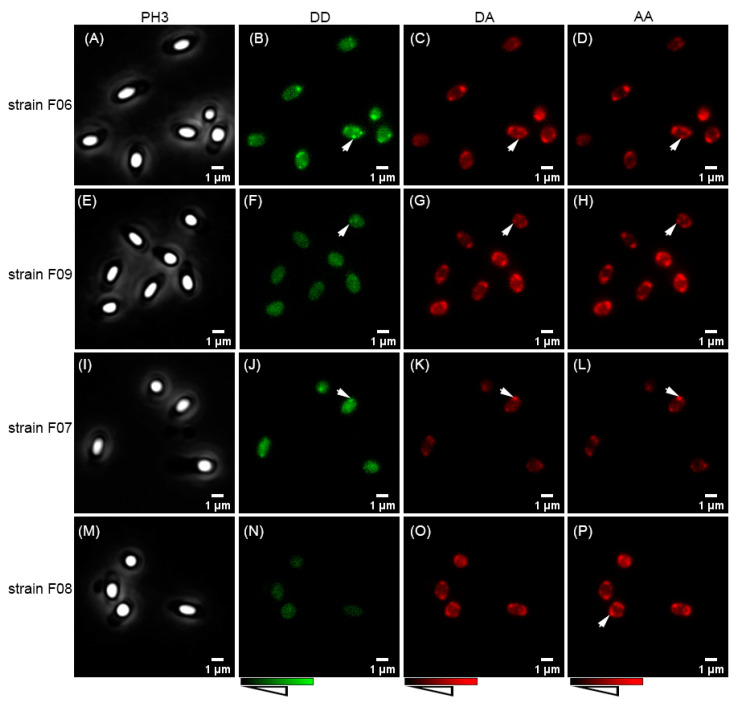
Images of dormant spores of *B. cereus* strains F06–F09. Panels (**A**–**D**)—GerRA-C-B–SGFP2 and GerD–mScarlet-I independently expressed in spores of strain F06. Panels (**E**–**H**)—GerRB-SGFP2 and GerD-mScarlet-I independently expressed in spores of strain F09. Panels (**I**–**L**)—GerRA–SGFP2 and GerD–mScarlet-I independently expressed in spores of strain F07. Panels (**M**–**P**)—GerRC–SGFP2 and GerD–mScarlet-I independently expressed in spores of strain F08. Column PH3 has phase-contrast images. Column DD has donor images at donor excitation. Column DA has acceptor channel images at donor excitation. Column AA has acceptor images at acceptor excitation. Arrows indicate putative germinosomes.

**Figure 5:**
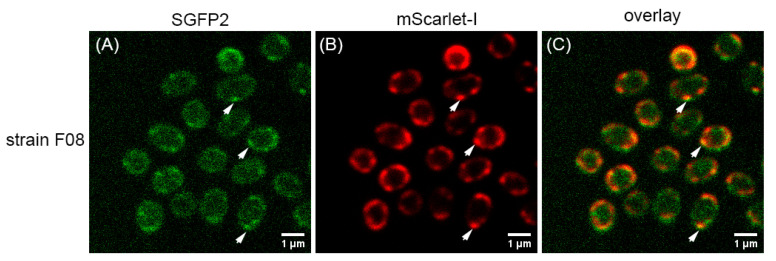
Images of dormant spores of *B. cereus* strain F08 by rescan confocal microscopy. (**A**) The green image was acquired with a GFP filter, (**B**) the red image was acquired with an RFP filter, and (**C**) the overlay image was generated from the merge of green and red images. The arrows show some GerRC–SGFP2 foci, the GerD–mScarlet-I foci, and the co-localized foci.

The membrane styryl dye FM 4-64 was used to stain the plasma membrane of *Escherichia coli* [30,31] and *B. subtills* spores’ IM [32,33,34]. Consequently, in the current work, the IM of developing *B. cereus* forespores of strains 006 expressing fusion protein GerR(A-C-B)–SGFP2, 010 expressing fusion protein GerD–SGFP2, and 012 expressing fusion protein SASP–SGFP2 were also stained with FM 4-64 (Figure 3A–C). As expected from previous results [15], there were one or two fluorescent foci outside the core in forespores of strains 006 and 010 (Figure 3(A-2),(B-2)). In contrast, strain 012 exhibited a bright fluorescent focus in the center of the forespore (Figure 3(C-2)), as expected, since *SASP* genes are expressed in the forespore and the gene products remain there as well [35]. Importantly, when the IM of forespores was visualized with FM 4-64 (Figure 3(A-3),(B-3),(C-3)), there were single foci in the overlay channel with strains 006 and 010 (Figure 3(A-4),(B-4)), but not with strain 012 (Figure 3(C-4)). Thus, as shown in the overlay channel, the fluorescent plot profiles created with ImageJ showed one peak, two peaks, and no peak in forespores of strains 006, 010, and 012, respectively (Figure 3(A-5),(B-5),(C-5)). These results indicated that germination proteins of the *gerR* operon and GerD were localized in putative germinosomes in the IM of developing forespores, while the SASP was only in the core or center of the forespore.

### 2.3. Interaction of GerR GR Proteins and GerD Assessed Using Spectral FRET Analyses

GerRB–SGFP2 expressed with GerRA and GerRC from plasmid pHT315-f06 was present in foci in spores of *B. cereus* strain F06 (Figure 4B) and in strain F09 in which only GerRB–SGFP2 was expressed (Figure 4F). Foci were also seen when GerRA–SGFP2 was expressed from plasmid pHT315-f07 in spores of strain F07 (Figure 4J). The fusion protein GerRC–SGFP2 was expressed from plasmid pHT315-f08 in spores of strain F08, but regular fluorescence microscopy did not show foci (Figure 4N). However, GerRC–SGFP2 foci were observed in spores of strain F08 by super resolution rescan confocal microscopy (Figure 5A). In addition, analysis of co-localization with 58 spores of strain F08 gave a Pearson’s coefficient of 0.511 between the GerRC–SGFP2 and GerD–mScarlet-I foci, suggesting these two fusion proteins are co-localized (Figure 5C). The GerD–mScarlet-I fusion protein was separately expressed from plasmids pHT315-f06, pHT315-09, pHT315-f07, and pHT315-f08 and gave foci in spores of all four of these strains (Figure 4D,H,L,P, and Figure 5B).

**Figure 6:**
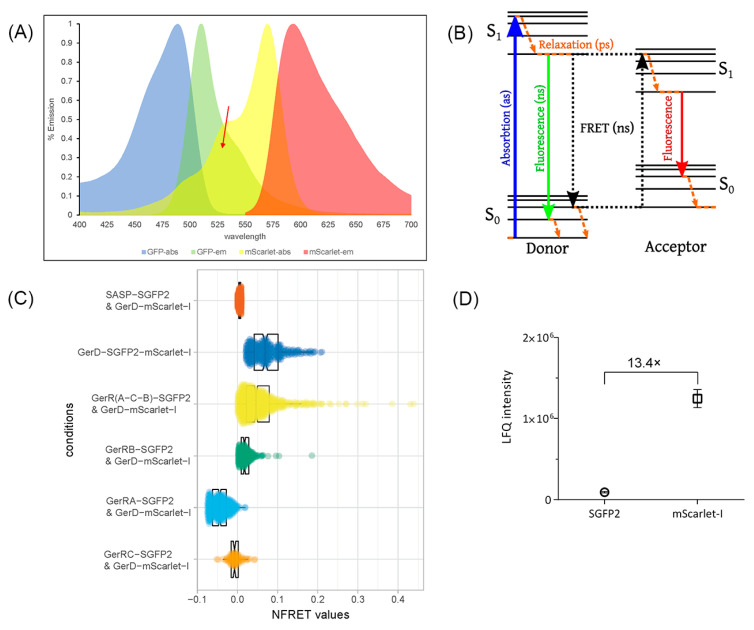
Analysis of interactions between IM proteins of the *gerR* operon and GerD by FRET. (**A**) The spectra of donor SGFP2 and acceptor mScarlet-I are shown. The specific wavelengths for excitation and emission are given in Table 2, in Materials and Methods. The red arrow indicates the spectral overlap between donor emission and acceptor excitation. (**B**) The FRET process is demonstrated in this Jablonski diagram (https://commons.wikimedia.org/w/index.php?curid=23197114, accessed on 30 June 2021). (**C**) Boxplots showing NFRET values in single spores of *B. cereus* strains F13 (SASP–SGFP2 and GerD–mScarlet-I), F11 (GerD–SGFP2–mScarlet-I), F06 (GerRB–SGFP2 and GerD–mScarlet-I), F09 (GerR(A-C-B)–SGFP2 and GerD–mScarlet-I), F07 (GerRA–SGFP2 and GerD–mScarlet-I), and F08 (GerRC–SGFP2 and GerD–mScarlet-I). The notches represent the 95% confidence interval for each median (data are given in Supplementary Materials Table S1). (**D**) Relative expression levels of SGFP2 and mScarlet-I in dormant spores of *B. cereus* strain F06 determined by mass spectrometry. The LFQ (label-free quantitation) intensity of SGFP2 and mScarlet-I indicated that the expression level of GerD was 13.4 times higher than each GerR protein. The sample processing, extraction of peptide material, and LC–FT–MS analysis were performed as described previously [36].

Interactions between GerR subunits and GerD in *B. cereus* spores of various strains were also examined by FRET (Figure 6A,B). For these analyses, SGFP2 as a donor was fused to the C-terminus of GerRB controlled by the native *gerR* operon’s promoter, and mScarlet-I as the acceptor was fused to the C-terminus of GerD controlled by the native *gerD* promoter. Previous work showed that SASP accumulates in the spore core [35], and as expected, the SASP–SGFP2 fusion protein was present in the core or center of dormant spores (Figure 3(C-2)). Because of this, we chose SASP as a biological negative control for the FRET analyses, and as expected, there was a 0% of FRET efficiency between SASP–SGFP2 and GerD–mScarlet-I in dormant spores of strain F13 (Figure 6C and Supplementary Materials Table S1). To obtain a biological positive control, the two reporter proteins, SGFP2 and mScarlet-I, were connected by a flexible linker and then fused to the C-terminus of GerD, so that the fusion protein GerD–SGFP2–mScarlet-I is in strain F11 spore’s IM. Importantly, there was 8% of FRET efficiency in the IM foci of spores of strain F11 (Figure 6C and Supplementary Materials Table S1).

We also used the native promoters of the *gerR* operon and *gerD* to independently express GerR(A-C-B)–SGFP2 and GerD–mScarlet-I from a plasmid in spores of strain F06. Mass spectrometry analysis of spore proteins showed that the proper fusion proteins were indeed expressed. The level of the GerD–mScarlet-I fusion protein was 13.4 times higher than that of GerR(A-C-B)–SGFP2 (Figure 6D). Additionally, there was 6% of FRET efficiency between GerR(A-C-B)–SGFP2 and GerD–mScarlet-I in spores of strain F06, as well between GerRB–SGFP2 and GerD–mScarlet-I in spores of strain F09 (Figure 6C and Supplementary Materials Table S1). However, the FRET efficiency calculations led to a small aberrant value of −4% between GerRA–SGFP2 and GerD–mScarlet-I in spores of strain F07 (Figure 6C and Supplementary Materials Table S1), although GerRA–SGFP2 does form foci in spores (Figure 4J). GerRC–SGFP2 also formed foci in spores, some of which co-localize with GerD–mScarlet-I (Figure 5), but only a small aberrant FRET value of −1% was observed between these two fluorescent proteins (Figure 6C and Supplementary Materials Table S1).

### 2.4. Dynamic Observation of Germinosome Formation during B. cereus Sporulation

**Figure 7:**
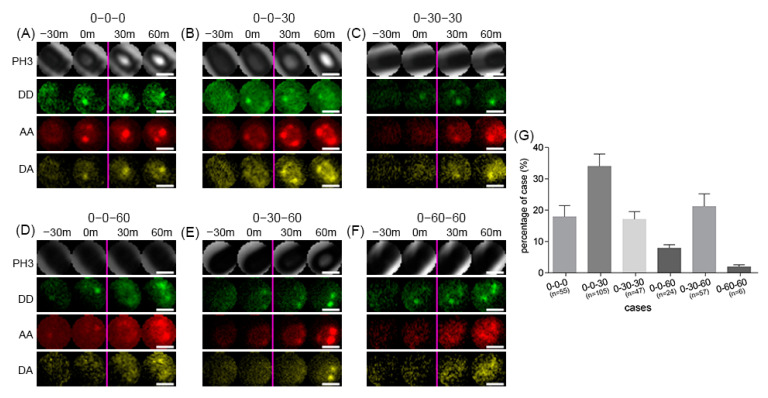
Dynamics of germinosome formation during forespore development in *B. cereus* strain F06. The time of appearance of DD foci for the first time-point is defined as time “0”, with subsequent images taken 30 and 60 min later in each row. The categories were defined according to the first appearance of foci in the DD, AA, and DA channels. (**A**) category 0-0-0, (**B**) category 0-0-30, (**C**) category 0-30-30, (**D**) category 0-0-60, (**E**) category 0-30-60, and (**F**) category 0-60-60. (**G**) Percentages of different categories. Abbreviations: PH3, phase-contrast; DD, donor image; AA, acceptor image; DA, FRET image. Scale bar = 1 µm.

Having shown close interaction between GerR(A-C-B)–SGFP2 and GerD–mScarlet-I in spores of *B. cereus*, using FRET (Figure 6C), we then turned to analysis of the dynamics of germinosome formation by time-lapse microscopy. To visualize single spores during sporulation, four-time points were selected (Figure 7). Initially, we found when GerR(A-C-B)–SGFP2 (in DD images) foci first appeared, and we defined this time as time “0”, relative to other time points. The rule for assigning stages for foci in each single spore was defined with a three-value term. The first, second, and third values are the time points of the first appearance in a DD image, an AA image, and a DA image for an individual spore, respectively. The results identified six categories of sporulating cells: (a) 0-0-0, DD, AA, and DA foci appeared at the same time-point (Figure 7A); (b) 0-0-30, DD and AA foci appeared at the same time point, but the appearance of DA foci was delayed 30 min (Figure 7B); (c) 0-30-30, DA and AA foci appeared at the same time point, but 30 min later than DD appearance (Figure 7C); (d) 0-0-60 was similar to category (b), but the appearance of the DA foci were delayed 60 min (Figure 7D); (e) 0-30-60, DD, AA, and DA foci were observed sequentially (Figure 7E); (f) 0-60-60 was similar to category (c), but the appearance of the DA and AA foci were delayed 60 min (Figure 7F). Notably, germinosome formation, as shown by FRET appearance, always preceded the appearance of phase-bright spores.

Calculation of the percentages of forespores in the different categories described above showed four major situations (Figure 7G). First, the DD and AA foci appeared simultaneously in 60% of *B. cereus* sporulating cells (categories 0-0-0, 0-0-30, and 0-0-60). Second, the AA and DA images appeared simultaneously in 37% of *B. cereus* sporulating cells (categories 0-0-0, 0-30-30, and 0-60-60). Third, the DD and AA foci appeared simultaneously followed by a 30 min delay before the DA image appeared in 34% of sporulating cells (category 0-0-30), and a 60 min delay in 8% of sporulating cells (category 0-0-60). Finally, the DD, AA and DA foci all appeared sequentially in 21% of sporulating cells (category 0-30-60). Overall, these results indicate that not all germinosome assemblies exhibit the same sequence of events, although germinosome formation always preceded the appearance of phase-bright spores. The results also indicated that foci of GerR(A-C-B)–SGFP2 and GerD–mScarlet-I can form simultaneously, but the appearance of an interaction signal from the FRET channel can be significantly delayed.

## 3. Discussion

In this study, we examined the locations of and interactions between the GR GerR subunits and GerD and recorded the dynamics of formation of a germinosome protein complex in the *B. cereus* spore IM. Our recent work showed that GerR subunits and GerD were well expressed, using their native promoters from plasmids in *B. cereus*, and could be easily visualized as fluorescent proteins [15]. The observed germinosome stability also suggests functionality for the SGFP2 and mScarlet-I proteins used. DNA FISH was used in the current work to show that almost all spores carry at least one copy of the plasmids used for expression of the fusion proteins, in agreement with previous work [15]. As expected, the fusion proteins expressed from the plasmid encoded genes were localized to the IM, as shown by the co-localization of the fusion proteins and the membrane dye FM 4-64. Co-localization analyses were performed for the various fusion proteins and possible close interaction between GerR subunits and GerD were studied by using FRET.

Previous work has shown that the C-terminus of *B. subtilis* GR A and C subunits and GerD are on the outer surface of spores’ IM [37]; the C-terminus of the B subunit of the *B. subtilis* GerA GR was also placed on the outside of spores’ IM based on modeling results [38]. Consequently, the predicted structure of these proteins suggests that the C-termini of GR subunits and GerD will all be on the outer surface of the IM in *B. subtilis* spores and by inference also most likely in *B. cereus* spores. In this study, we observed that the GerR and GerD fusion proteins were present as co-localized foci in dormant *B. cereus* spores and that GerRB–SGFP2 expressed with or without GerRA and GerRC, both gave normalized FRET with GerD–mScarlet−I. In contrast, the A subunit showed no positive FRET signal with GerD. Notice that, while GerRA–SGFP2 foci were detected by wide-field fluorescence microscopy, because of low expression levels, the GerRC–SGFP2 foci could only be detected by using rescan confocal microscopy. However, this GR subunit also gave no positive FRET with GerD. A surprising result in the FRET work was the normalized FRET values close to zero of −0.04 and −0.01 for the GerRA−SGFP2 GerD−mScarlet-I and GerRC-SGFP2 GerD-mScarlet-I donor/acceptor pair, respectively. One possible reason for this result is that GerRA-SGFP2 was expressed from plasmid in developing spores of *B. cereus* strain F07 at a relatively high level, leading to high fluorescence intensity, as well as a wide range of intensity distribution in single spores. Both results were in comparison to GerRB-SGFP2 levels in cells with or without co-expression of GerRA and GerRC (Supplementary Materials Figure S1). The higher expression levels of GerRA–SGFP2 in strain F07 may be due to the fact that the A subunit is the first gene encoded by the *gerR* operon. Other possibilities are that GerRA–SGFP2 alone is more stable by itself than GerRB–SGFP2 alone and that the environment of the SGFP2 in GerRA–SGFP2 may be very different from that of the SGFP2 fused to GerRB, possibly leading to higher fluorescence. The latter could also affect the FRET measurements between GerD–mScarlet-I with GerRA–SGFP2. In addition, the fluorescence intensity of GerRC–SGFP2 in strain F08 is significantly lower than GerRA–SGFP2 in strain F07 or GerR(A-C-B)–SGFP2 in strain F06 (Supplementary Materials Figure S1). Perhaps less of the C subunit is tightly associated in the GerR GR. Furthermore, there is a very different IM association of GerRC compared to GerRA and GerRB, as, in contrast to GerRA and GerRB, which have integral membrane protein modules, the GerRC subunit is held in the IM only by a lipid anchor. However, the most important conclusion from the FRET work was that there is close contact (<10 nm) between the B subunit of the GerR GR and GerD, and not between GerD and either GerRA or GerRC subunits. Indeed, previous work found no interaction in vitro between GerD and a GR C subunit [25]. We conclude that the assembly of GRs in a germinosome requires close interaction (<10 nm) between GerD and GR B subunits in *B. cereus* spores, and thus likely in all *Bacillus* spore formers. Further experiments may be performed, i.e., the knockout or silencing of the chromosomal *gerR* operon and *gerD* gene, to avoid competition with chromosomal genes in studying the assembly dynamics of germinosomes in more detail. In addition, there are more techniques based on the FRET principle, for example, acceptor photobleaching, acceptor photoswitching by confocal microscopy, or a combination with fluorescence lifetime imaging microscopy (FLIM–FRET) to minimize the sensitivity of the results with respect to differences in the concentration of donor and acceptor [39,40,41,42]. As shown, our data indicate that here the relative amount of mScarlet-I (fluorophore of the GerD acceptor) was significantly (~13 times) higher than SGFP2 (the fluorophore of the GR-GerR donor).

We also used the plasmid-expressed fusion proteins GerR(A-C-B)–SGFP2 and GerD–mScarlet-I to observe the dynamics of germinosome formation in *B. cereus*. Previous work indicates that GerD has an important role in nutrient germination, most likely by coalescing GRs in a germinosome in *B. subtilis* spores [19]. However, the formation of this complex between GRs and GerD during sporulation has never been studied, not even in *B. subtilis*. Previous work has shown that *B. subtilis* cell populations exhibit significant heterogeneity in progression through sporulation, particularly in the transition from phase-dark to phase-bright spores [43], and this was also seen in the current work with sporulating *B. cereus*. In addition, not all assembly of *B. cereus* germinosomes followed the same trajectory, as the GerR focus formed before the GerD focus in some forespores, suggesting that either GerR proteins alone can at least transiently form an IM focus, or that even low GerD levels can nucleate GerR focus formation. Our work also showed that the appearance of GerR(A-C-B)–SGFP2–GerD–mScarlet-I foci with good FRET between GerRB and GerD was always observed before the appearance of phase-bright spores.

In summary, the GR GerR and GerD were localized in the IM of *B. cereus* spores, and positive normalized FRET data indicated that the GerR B subunit (but not the A or C subunits) and GerD were less than 10 nm from one another. Studying the dynamics of the sporulation process showed that GerR(A-C-B)–SGFP2 and GerD–mScarlet-I co-localization often preceded the GerRB–GerD close interaction, but the latter always preceded the appearance of the phase-bright spores. Further studies along these lines, with other key components of the spore-germination machinery, i.e., proteins of the SpoVA channel, will be instrumental in unraveling the molecular basis of the dynamics of spore formation and germination in Bacilli, including the mode of action of GRs in the pathogenic food-related bacterium *B. cereus*.

## 4. Materials and Methods

### 4.1. Construction of B. cereus Strains with Plasmids Expressing Various Proteins

In this study, the genes of interest were the *gerR* operon genes *gerRA* (BC0784), *gerRC* (BC0783), and *gerRB* (BC0782); the *gerD* gene (BC0169); and a gene encoding a *SASP* (BC4646). Gene numbers refer to the genome of *B. cereus* ATCC 14579 (GenBank: AE016877). Promoter regions of these genes were selected to be in the ~600 bp upstream from the genes’ translation start sites, as has been shown for GR operons, *gerD*, and SASP genes in *B. cereus* [44], and these regions were separately amplified from genomic DNA of *B. cereus* ATCC 14579 with the primers in Supplementary Materials Table S2. The promoter regions chosen are 664 bp upstream of the *gerR* operon, 606 bp upstream of *gerD*, and 578 bp upstream of the SASP gene, and named PR, PD, and PS, respectively. The *SGFP2* fragment with stop codons was separately fused to the 3′ end of the *gerR(A-C-B), gerRA*, *gerRB*, *gerRC*, *gerD,* and *SASP* genes, using a two-step fusion PCR, and the fusion fragment was inserted into pHT315 between *Eco*R I and *Hin*d III sites (Table 1). This ligation product was transformed into competent *E. coli* DH5α cells [45], and selection of positive clones resulted in plasmids pHT315-f01, pHT315-f02, pHT315-f03, pHT315-f04, pHT315-10, and pHT315-f12 (Table 1 and Figure 1B). The *mScarlet-I* fragment with stop codons was fused to the 3′ end of *gerD* with a two-step fusion PCR, and the resulting fusion fragment was inserted into pHT315 between *Kpn* I and *Eco*R I sites, and termed pHT315-f05 (Table 1 and Figure 1B). Plasmid pHT315-f05 was also used as a backbone to construct a plasmid independently expressing two fusion genes. The fusion fragments PR-*gerRA-RC-RB-SGFP2*, PR-*gerRA-SGFP2*, PR-*gerRC-SGFP2*, PR-*gerRB-SGFP2*, or PS-*SASP-SGFP2* were separately inserted into pHT315-f05 between the *Kpn* I and *Hin*d III sites, resulting in pHT315-f06, pHT315-f07, pHT315-f08, pHT315-f09, and pHT315-f13, respectively (Table 1 and Figure 1C). The correct construction of all recombinant plasmids was confirmed by using DNA sequencing (Macrogen Europe B.V., Amsterdam, The Netherlands). All plasmids having the correct sequence were introduced into competent cells of *B. cereus* ATCC 14579 by electroporation [15]. The transformation mixture was plated on tryptic soy agar with 10 µg/mL erythromycin and incubated overnight at 30 °C. On the following day, an erythromycin-positive single colony was analyzed by colony PCR, using sequencing primers 315_YW35 and 315_YW36 (Supplementary Materials Table S1). Then, based on our previous work, the colonies giving PCR fragments of the correct size were used to prepare sporulating cells and to prepare and purify dormant spores [15].

### 4.2. Fluorescence In Situ Hybridization for DNA

The procedure for fluorescence in situ hybridization (FISH) for DNA was modified from a published method [46]. The DNA-FISH probe was designed by labeling a fragment of the erythromycin resistance gene (*erm*) in *B. cereus* spores. The DNA FISH probe was amplified from plasmid pHT315, using the forward primer (5′–ACG ACG AAA CTG GCT AAA A–3′) and reverse primer (5′–TCT GTG GTA TGG CGG GTA A-3′). The purified PCR product of 413 bp was labeled with orange 552-dUTP, using the Nick translation DNA-labeling system 2.0 (Enzo Life Sciences BVBA, Bruxelles, Belgium), and this was followed by ethanol precipitation with 3 µg human Cot DNA, which is used to block non-specific hybridization [47] in 0.1× total volume of 3 M sodium acetate pH 5.2 plus 2.5× total volume of ice-cold absolute ethanol; the mix was vortexed and incubated for 30 min at, −20 °C. After centrifugation (17,000× *g* at 4 °C for 30 min), the DNA pellet was suspended in nuclease-free water with 2.33 volumes of mixed hybridization buffer (50% formamide (Sigma-Aldrich, Amsterdam, The Netherlands), 10% dextran sulfate (Sigma-Aldrich, Amsterdam, The Netherlands), 2× SSC (20× Saline Sodium Citrate Buffer, Sigma-Aldrich, Amsterdam, The Netherlands), and 1% Tween-20), giving a probe working solution.

Spores (1 mL, OD600 = 2) were briefly centrifuged and treated with 500 µL 50% ethanol for 30 min at 80 °C in a heat block, followed by brief centrifugation and pellets were suspended in 500 µL SDS/DTT solution (10 mg/mL SDS, 50 mM DTT) and incubated for 30 min at 65 °C. After washing 3 times with Milli-Q water, the pellet was suspended in 100 µL Milli-Q water. The treated spores (30 µL) were dropped onto a poly-L-lysine-coated glass slide (Sigma-Aldrich, Amsterdam, The Netherlands). After air-drying, the slide was covered with 10 mg/mL lysozyme solution for 30 min, at 37 °C; washed twice with Milli-Q water for 5 min; and the slide was dehydrated in 50, 80, and finally 100% ethanol for 3 min each. The slide was then covered with hybridization buffer with probe working solution, following denaturation for 3 min at 75 °C, and incubated overnight, at 37 °C. Subsequently, the slide was immersed in 0.4× SSC for 5 min, at 75 °C; in 4× SSC with 0.1% Tween-20 for 2 min, at 23 °C; and then in Milli-Q water for 2 min. The specifically bound probes were visualized by wide field florescence microscopy with excitation at 555 nm and emission at 576 nm. Images were analyzed with Fiji/ImageJ software.

### 4.3. FM 4-64 Staining, Visualization, and Image Analysis

Sporulation and the preparation of chemically defined growth and sporulation (CDGS) medium were carried out as described previously [15]. At the 48th h of sporulation, 1.8 mL of culture was centrifuged at 2400× *g* for 6 min, suspended in 1 mL of CDGS medium containing FM 4-64 at 2.5 µg/mL, and the mixture shaken at 30 °C for 50 min, at 150 rpm. After this staining, the culture was centrifuged briefly and suspended in fresh CDGS medium to an OD600 of 15~20, and this culture was used for further imaging.

Preparation of microscope slides and imaging were as previous described [15,48]. The visualization of forespores of *B. cereus* strain 006 expressing GerR(A-C-B)–SGFP2, forespores of *B. cereus* strain 010 expressing GerD–SGFP2, and forespores of *B. cereus* strain 012 expressing SASP–SGFP2 was carried out with excitation at 470 ± 12 nm and emission at 516 ± 24 nm. FM 4-64 was visualized with excitation at 555 nm and emission at 630 nm. All microscope images were analyzed with Fiji/ImageJ software (http://rsbweb.nih.gov/ij, accessed on 30 June 2021).

### 4.4. Microscopy Settings and Data Analysis of FRET

A Nikon Eclipse Ti-E microscope (Nikon Instruments, Tokyo, Japan) equipped with a sCmos camera (Hamamatsu Flash 4.0 V2, Hamamatsu City, Japan), phase-contrast, and wide-field fluorescence components was used to detect the sensitized emission FRET, using fluorescent reporter proteins SGFP2 and mScarlet-I in dormant spores of *B. cereus*, as previously described [15,42,49,50]. Briefly, the spectral bleed-through (BT) and cross-excitation (CE) factors of donor or acceptor were determined with spores expressing only SGFP2 or mScarlet-I. Three images were captured from each spore by microscopy (Table 2), the donor image (*I_DD_* image) acquired with donor excitation and donor emission, an acceptor image (*I_AA_* image) acquired with acceptor excitation and acceptor emission, and a sensitized fluorescence FRET image (*I_DA_* image) acquired with donor excitation and acceptor emission. The acquired 16-bit type images of the *I_DD_, I_AA_*_,_ and *I_DD_* were converted to 32-bit type, and then selection and measurement of the area of background without samples of images were performed, following background subtraction by using the tool Process—Math—Subtract in Fiji/ImageJ. The fluorescence intensity of each spore was measured by the ObjectJ SporeAnalyzer_1c.ojj in Fiji/ImageJ (https://sils.fnwi.uva.nl/bcb/objectj/examples/SporeAnalyzer/MD/SporeAnalyzer.html, accessed on 14 June 2021). Fluorescence intensities of plasmid-containing spores that were lower than that of wild-type spores were considered as aberrant values and were not counted. The BT values of donor and acceptor were calculated with fluorescence intensity using linear relationships. In this work, we used different fusion proteins as the donor and the same fusion protein as the acceptor; the acceptor also looks more stable than the donor in different spores. Normalized FRET (NFRET) values were generated by the fluorescence intensities in acceptor images from the formula below, and NFRET values of single spores were presented in a boxplot [51].

(1) $$NFRET=\frac{I_{DA}-BT_{donor}\times I_{DD}-BTCE_{acceptor}\times I_{AA}}{I_{AA}}$$

**Table 2:** Wavelengths of the microscope excitation and emission filters used in this study.

Images	Excitation (nm)	Emission (nm)
Donor image	Donor (458–483)	Donor (492–541)
FRET image	Donor (458–483)	Acceptor (570–616)
Acceptor image	Acceptor (543–558)	Acceptor (570–616)

Spores of strain F08 expressing the fusion proteins GerRC–SGFP2 and GerD–mScarlet-I were also analyzed by a Nikon Eclipse Ti-E microscope (Nikon Instruments, Tokyo, Japan)equipped with a super-resolution rescan confocal module (home build), EMCCD camera (Andor iXon Ultra 888, Oxford Instruments, Abingdon, UK), and 100x objective (CFI Apo TIRF 100X Oil, NA 1.49, Nikon Instruments, Tokyo, Japan). The SGFP2 images were captured for 3 s, at a laser power of 5%, at an excitation of 488 nm, and at an emission of 500–550 nm (Semrock FF03-525/50-25, IDEX Corporation, based in Northbrook, Lake Forest, IL, USA). The mScarlet-I images were captured for 3 s, at a laser power of 3%, with excitation at 561 nm and emission at 572 nm long-pass (HQ572LP, Chroma Technology Corporation, Bellows Falls, VT, USA). The analysis of co-localization was carried out by the plug-in JACoP used to calculate the co-localization indicator, usually a Pearson’s coefficient [52].

### 4.5. Time-Lapse Settings and Data Analysis in B. cereus Sporulation

*B. cereus* strain F06 expressing GerR(A-C-B) fused to SGFP2 and GerD fused to mScarlet-I was used to study the dynamics of germinosome formation, using wide-field fluorescence microscopy. The microscope slides were prepared as previously described [48], but one difference was that the agarose pad contained 1% agarose and 0.1× CDGS medium. At an OD600 of ~0.5, a vegetative culture was centrifuged briefly and suspended in 0.2× CDGS medium to an OD600 of ~10. Then, 1.3 µL of the mixture was pipetted on the 1% agarose pad and made ready to be followed and recorded. Microscopy time-lapse images were recorded at 30 min intervals for 72 h at 30 °C. Data analysis used the ObjectJ plugin Sporulation_1i.ojj (https://sils.fnwi.uva.nl/bcb/objectj/examples/Sporulation/MD/Sporulation.html, accessed on 29 June 2021). Three independent experiments were performed, and the data were analyzed by software GraphPad 6.0 and expressed as mean ± SEM.

## Data Availability

Raw data is available from the corresponding author upon motivated request.

## Supplementary Materials

The following are available online at https://www.mdpi.com/article/10.3390/ijms222011230/s1. Table S1. Table S2. Figure S1.

## References

[1] Blakey L.J., Priest F.G. (1980). The occurrence of *Bacillus cereus* in some dried foods including pulses and cereals. J. Appl. Bacteriol..

[2] Stenfors Arnesen L.P., Fagerlund A., Granum P.E. (2008). From soil to gut: *Bacillus cereus* and its food poisoning toxins. FEMS Microbiol. Rev..

[3] Bottone E.J. (2010). *Bacillus cereus*, a volatile human pathogen. Clin. Microbiol. Rev..

[4] Dréan P., McAuley C.M., Moore S.C., Fegan N., Fox E.M. (2015). Characterization of the spore-forming *Bacillus cereus* sensu lato group and *Clostridium perfringens* bacteria isolated from the Australian dairy farm environment. BMC Microbiol..

[5] Khanna K., Lopez-Garrido J., Zhao Z., Watanabe R., Yuan Y., Sugie J., Pogliano K., Villa E. (2019). The molecular architecture of engulfment during *Bacillus subtilis* sporulation. eLife.

[6] Higgins D., Dworkin J. (2012). Recent progress in *Bacillus subtilis* sporulation. FEMS Microbiol. Rev..

[7] Sonenshein A.L. (2000). Control of sporulation initiation in *Bacillus subtilis*. Curr. Opin. Microbiol..

[8] Setlow P. (2007). I will survive: DNA protection in bacterial spores. Trends Microbiol..

[9] Bressuire-Isoard C., Bornard I., Henriques A., Carlin F., Broussolle V. (2016). Sporulation temperature reveals a requirement for CotE in the assembly of both the coat and exosporium layers of *Bacillus cereus* spores. Appl. Environ. Microbiol..

[10] Giorno R., Bozue J., Cote C., Wenzel T., Moody K.-S., Mallozzi M., Ryan M., Wang R., Zielke R., Maddock J.R. (2007). Morphogenesis of the *Bacillus anthracis* spore. J. Bacteriol..

[11] Setlow P. (2014). Spore resistance properties. Microbiol. Spectr..

[12] Rudner D.Z., Pan Q., Losick R.M. (2002). Evidence that subcellular localization of a bacterial membrane protein is achieved by diffusion and capture. Proc. Natl. Acad. Sci. USA.

[13] Wen J., Pasman R., Manders E.M., Setlow P., Brul S. (2019). Visualization of germinosomes and the inner membrane in *Bacillus subtilis* spores. J. Vis. Exp..

[14] Paidhungat M., Setlow P. (2001). Localization of a germinant receptor protein (GerBA) to the inner membrane of *Bacillus subtilis* spores. J. Bacteriol..

[15] Wang Y., De Boer R., Vischer N., Van Haastrecht P., Setlow P., Brul S. (2020). Visualization of germination proteins in putative *Bacillus cereus* germinosomes. Int. J. Mol. Sci..

[16] Hudson K.D., Corfe B.M., Kemp E.H., Feavers I.M., Coote P.J., Moir A. (2001). Localization of GerAA and GerAC germination proteins in the *Bacillus subtilis* spore. J. Bacteriol..

[17] Paredes-Sabja D., Setlow P., Sarker M.R. (2011). Germination of spores of *Bacillales* and *Clostridiales* species: Mechanisms and proteins involved. Trends Microbiol..

[18] Pelczar P.L., Setlow P. (2008). Localization of the germination protein GerD to the inner membrane in *Bacillus subtilis* spores. J. Bacteriol..

[19] Pelczar P.L., Igarashi T., Setlow B., Setlow P. (2007). Role of GerD in germination of *Bacillus subtilis* spores. J. Bacteriol..

[20] Vepachedu V.R., Setlow P. (2005). Localization of SpoVAD to the inner membrane of spores of *Bacillus subtilis*. J. Bacteriol..

[21] Valdespino A.P., Li Y., Setlow B., Ghosh S., Pan D., Korza G., Feeherry F.E., Doona C.J., Hao B., Setlow P. (2014). Function of the SpoVAEa and SpoVAF proteins of *Bacillus subtilis* spores. J. Bacteriol..

[22] Setlow P. (2003). Spore germination. Curr. Opin. Microbiol..

[23] Hornstra L.M., de Vries Y.P., de Vos W.M., Abee T., Wells-Bennik M.H.J. (2005). *gerR*, a novel ger operon involved in _L_-alanine- and inosine-initiated germination of *Bacillus cereus* ATCC 14579. Appl. Environ. Microbiol..

[24] Ross C., Abel-Santos E. (2010). The ger receptor family from sporulating bacteria. Curr. Issues Mol. Biol..

[25] Li Y., Setlow B., Setlow P., Hao B. (2010). Crystal structure of the GerBC component of a *Bacillus subtilis* spore germinant receptor. J. Mol. Biol..

[26] Griffiths K.K., Zhang J., Cowan A.E., Yu J., Setlow P. (2011). Germination proteins in the inner membrane of dormant *Bacillus subtilis* spores colocalize in a discrete cluster. Mol. Microbiol..

[27] Kremers G.-J., Goedhart J., van den Heuvel D.J., Gerritsen H.C., Gadella T.W. (2007). Improved green and blue fluorescent proteins for expression in bacteria and mammalian cells. Biochemistry.

[28] Bindels D.S., Haarbosch L., van Weeren L., Postma M., Wiese K.E., Mastop M., Aumonier S., Gotthard G., Royant A., Hink M.A. (2017). mScarlet: A bright monomeric red fluorescent protein for cellular imaging. Nat. Methods.

[29] Carrera M., Zandomeni R., FitzGibbon J., Sagripanti J.-L. (2007). Difference between the spore sizes of *Bacillus anthracis* and other *Bacillus* species. J. Appl. Microbiol..

[30] Fishov I., Woldringh C.L. (1999). Visualization of membrane domains in *Escherichia coli*. Mol. Microbiol..

[31] Lim G.E., Derman A.I., Pogliano J. (2005). Bacterial DNA segregation by dynamic SopA polymers. Proc. Natl. Acad. Sci. USA.

[32] Pogliano J., Osborne N., Sharp M.D., Mello A.A.-D., Perez A., Sun Y.-L., Pogliano K. (1999). A vital stain for studying membrane dynamics in bacteria: A novel mechanism controlling septation during *Bacillus subtilis* sporulation. Mol. Microbiol..

[33] Cowan A.E., Olivastro E.M., Koppel D.E., Loshon C.A., Setlow B., Setlow P. (2004). Lipids in the inner membrane of dormant spores of *Bacillus species* are largely immobile. Proc. Natl. Acad. Sci. USA.

[34] Omardien S., Drijfhout J.W., Zaat S.A., Brul S. (2018). Cationic amphipathic antimicrobial peptides perturb the inner membrane of germinated spores thus inhibiting their outgrowth. Front. Microbiol..

[35] Setlow P. (1988). Small, acid-soluble spore proteins of *Bacillus* species: Structure, synthesis, genetics, function, and degradation. Annu. Rev. Microbiol..

[36] Swarge B.N., Roseboom W., Zheng L., Abhyankar W., Brul S., De Koster C.G., De Koning L.J. (2018). “One-pot” sample processing method for proteome-wide analysis of microbial cells and spores. Proteom. Clin. Appl..

[37] Korza G., Setlow P. (2013). Topology and accessibility of germination proteins in the *Bacillus subtilis* spore inner membrane. J. Bacteriol..

[38] Blinker S., Vreede J., Setlow P., Brul S. (2021). Predicting the structure and dynamics of membrane protein GerAB from *Bacillus subtilis*. Int. J. Mol. Sci..

[39] Alexeeva S., Gadella T.W.J., Verheul J., Verhoeven G.S., Blaauwen T.D. (2010). Direct interactions of early and late assembling division proteins in *Escherichia coli* cells resolved by FRET. Mol. Microbiol..

[40] Becker W. (2012). Fluorescence lifetime imaging—techniques and applications. J. Microsc..

[41] Qian J., Yao B., Wu C. (2014). Fluorescence resonance energy transfer detection methods: Sensitized emission and acceptor bleaching. Exp. Ther. Med..

[42] Rainey K.H., Patterson G.H. (2019). Photoswitching FRET to monitor protein—protein interactions. Proc. Natl. Acad. Sci. USA.

[43] Kearns D. (2005). Cell population heterogeneity during growth of *Bacillus subtilis*. Genes Dev..

[44] Ghosh A., Manton J.D., Mustafa A.R., Gupta M., Ayuso-Garcia A., Rees E.J., Christie G. (2018). Proteins encoded by the *gerP* operon are localized to the inner coat in *Bacillus cereus* spores and are dependent on GerPA and SafA for assembly. Appl. Environ. Microbiol..

[45] Tang X., Nakata Y., Li H.-O., Zhang M., Gao H., Fujita A., Sakatsume O., Ohta T., Yokoyama K. (1994). The optimization of preparations of competent cells for transformation of *E. coli*. Nucleic Acids Res..

[46] Greenberg E., Hochberg-Laufer H., Blanga S., Kinor N., Shav-Tal Y. (2019). Cytoplasmic DNA can be detected by RNA fluorescence In Situ hybridization. Nucleic Acids Res..

[47] Eykelenboom J.K., Gierliński M., Yue Z., Hegarat N., Pollard H., Fukagawa T., Hochegger H., Tanaka T.U. (2019). Live imaging of marked chromosome regions reveals their dynamic resolution and compaction in mitosis. J. Cell Biol..

[48] Pandey R., Ter Beek A., Vischer N.O.E., Smelt J.P.P.M., Brul S., Manders E.M.M. (2013). Live cell imaging of germination and outgrowth of individual *Bacillus subtilis* spores; the effect of heat stress quantitatively analyzed with SporeTracker. PLoS ONE.

[49] Xia Z., Liu Y. (2001). Reliable and global measurement of fluorescence resonance energy transfer using fluorescence microscopes. Biophys. J..

[50] Yang Y., Gaspard G., McMullen N., Duncan R. (2020). Polycistronic genome segment evolution and gain and loss of FAST protein function during fusogenic orthoreovirus speciation. Viruses.

[51] Postma M., Goedhart J. (2019). PlotsOfData—a web app for visualizing data together with their summaries. PLoS Biol..

[52] Bolte S., Cordelières F. (2006). A guided tour into subcellular colocalization analysis in light microscopy. J. Microsc..

