# Supplementary material for: Dynamics of Germinosome Formation and FRET-Based Analysis of Interactions between GerD and Germinant Receptor Subunits in Bacillus cereus Spores
Source: Int J Mol Sci. 2021 Oct 18;22(20):11230. doi: 10.3390/ijms222011230 (PMC8539644; doi:10.3390/ijms222011230)
Supplement: Supplementary file 1 [file ijms-22-11230-s001.zip › ijms-1395443-supplementary.pdf]

Dynamics of germinosome formation and FRET-based analysis of interactions between GerD and germinant receptor subunits in *Bacillus cereus* spores

Supplementary Materials

Table S1. Average FRET efficiency in individual spores of various strains normalized by donor channel as described in Methods.

Fusion protein(s) from plasmid*		Names of plasmid	Number of spores	mean	95%Cl Mean
**negative control**					
SASP-SGFP2	GerD-mScarlet-I	pHT315-f13	191	0	0 ~ 0
**positive control**					
GerD-SGFP2-mScarlet-I		pHT315-f11	237	0.08	0.07 ~ 0.08
**interaction**					
GerR(A-C-B)-SGFP2	GerD-mScarlet-I	pHT315-f06	489	0.06	0.06 ~ 0.07
GerRB-SGFP2	GerD-mScarlet-I	pHT315-f09	179	0.02	0.02 ~ 0.02
GerRA-SGFP2	GerD-mScarlet-I	pHT315-f07	323	-0.04	-0.05 ~ -0.04
GerRC-SGFP2	GerD-mScarlet-I	pHT315-f08	81	-0.01	-0.01 ~ 0

*A shuttle vector originally for *E. coli* and *B. thuringiensis*.

Table S2. Primers used in this study

primers	sequence	purpose or function
315_YW1	CGGGGTACCCACCTTCCCTATATCCGCT	*Kpn* I-*gerR* promoter-Fw
315_YW2	CCGGAATTCCACCTTCCCTATATCCGCT	*Eco*R I-*gerR* promoter-Fw
315_YW3	CGGGATCCTCTCACCCCTAACATATATC	*Bam*H I-*gerR* promoter-Rv
315_YW4	CGGGATCCATGTTCGGTTTATCATCT	*Bam*H I-*gerRA*-Fw
315_YW5	CTCGCCCTTGCTCACCATgctgccgctgccgctgccTTCATTCTCACCATCACGTT	*gerRA-$(GS)_3$-SGFP2*-Rv
315_YW6	AACGTGATGGTGAGAATGAAggcagcggcagcggcagcATGGTGAGCAAGGGCGAG	*gerRA-$(GS)_3$-SGFP2*-Fw
315_YW7	CGGGATCCGAATGAATGAAGAAAATTTTA	*Bam*H I-*gerRC*-Fw
315_YW8	CTCGCCCTTGCTCACCATgctgccgctgccgctgccTTCCCCGATTCCAGATTGT	*gerRC-$(GS)_3$-SGFP2*-Rv
315_YW9	ACAATCTGGAATCGGGGAAggcagcggcagcggcagcATGGTGAGCAAGGGCGAG	*gerRC-$(GS)_3$-SGFP2*-Fw
315_YW10	CGGGATCCTGAGGTGAAATGAGCAATGA	*Bam*H I-*gerRB*-Fw
315_YW11	CTCGCCCTTGCTCACCATgctgccgctgccgctgccAGGTGTATCGGTTGAAGA	*gerRB-$(GS)_3$-SGFP2*-Rv
315_YW12	TCTTCAACCGATACACCTggcagcggcagcggcagcATGGTGAGCAAGGGCGAG	*gerRB-$(GS)_3$-SGFP2*-Fw
315_YW13	CCCAAGCTTTTACTTGTACAGCTCGTCCAT	*SGFP2-Hin*d III-Rv

315_YW14	AATCTGGAATCGGGGAATAGTGAGGTGAAATGAGCAATG	*gerRC-gerRB*-Fw
315_YW15	CATTGCTCATTTCACCTCACTATTCCCCGATTCCAGATT	*gerRC-gerRB*-Rv
315_YW16	GCTCTAGAACAACCATAAAGAACAGAGC	*Xba* I-*gerD* promoter-Fw
315_YW17	TTCTCCTTTACTCACCATgctgccgctgccgctgccCTGTTCTTCCTTCTTCTCG	*gerD*-$(GS)_3$-*mScarlet-I*-Rv
315_YW18	CGAGAAGAAGGAAGAACAGggcagcggcagcggcagcATGGTGAGTAAAGGAGAA	*gerD*-$(GS)_3$-*mScarlet-I*-Fw
315_YW19	GCTCTAGATTATTTGTATAGTTCATCCAT	*mScarlet-I-Xba* I-Rv
315_YW20	CCGGAATTCTTATTTGTATAGTTCATCCAT	*mScarlet-I-Eco*R I-Rv
315_YW21	GCTCTAGACACCTTCCCTATATCCGCT	*Xba* I-*gerR* promoter-Fw
315_YW22	GCTGAAAGGAGTTCCAACTTATGAAACGGATGCTATT	*gerD* promoter-*gerD*-Fw
315_YW23	AATAGCATCCGTTTCATAAGTTGGAACTCCTTTCAGC	*gerD* promoter-*gerD*-Rv
315_YW24	GCATGGACGAGCTGTACAAGggcagcggcagcggcagcATGGTGAGTAAAGGAGAA	*SGFP2*-$(GS)_3$-*mScarlet-I*-Fw
315_YW25	TTCTCCTTTACTCACCATgctgccgctgccgctgccCTTGTACAGCTCGTCCATGC	*SGFP2*-$(GS)_3$-*mScarlet-I*-Rv
315_YW26	CCCAAGCTTTTATTTGTATAGTTCATCCAT	*mScarlet-I-Hind* III-Rv
315_YW27	GGGGTACCCTTCACCTTGTTCCGTTT	*Kpn* I-*SASP* promoter-Fw
315_YW28	CAATTAGGCGGTTACCAAAAggcagcggcagcggcagcATGGTGAGCAAGGGCGAG	*SASP*-$(GS)_3$-*SGFP2*-Fw
315_YW29	CTCGCCCTTGCTCACCATgctgccgctgccgctgccTTTTTGGTAACCGCCTAATTG	*SASP*-$(GS)_3$-*SGFP2*-Rv
315_YW30	GGGGTACCACAACCATAAAGAACAGAGC	*Kpn* I-*gerD* promoter-Fw
315_YW31	CTCGCCCTTGCTCACCATgctgccgctgccgctgccCTGTTCTTCCTTCTTCTCG	*gerD*-$(GS)_3$-*SGFP2*-Rv
315_YW32	CGAGAAGAAGGAAGAACAGggcagcggcagcggcagcATGGTGAGCAAGGGCGAG	*gerD*-$(GS)_3$-*SGFP2*-Fw
315_YW33	CTCTTCGCTATTACGCCA	T vector-sequencing-Fw
315_YW34	TCACACAGGAAACAGCTA	T vector-sequencing-Rv
315_YW35	ATGTTGTGTGGAATTGTGAG	315-sequencing-Fw
315_YW36	AAGGCGATTAAGTTGGGT	315-sequencing-Rv
315_YW37	CAAAGACCCCAACGAGAA	*SGFP2*-sequencing-Fw
315_YW38	GCTGGGGAAAATAAGTATGA	*gerRC*-sequencing-Fw
315_YW39	GCGCTACAATCCAAACAAA	*gerRA*-sequencing-Fw
315_YW40	CCGGAATTCATGAAACGGATGCTATTC	*Eco*R I-*gerD*-Fw
315_YW41	CGTTTTGGACTAGGGATG	*gerD* promoter-sequencing-Rv

a. Restriction cleavage sites are underlined.

b. The flexible linker $(GS)_3$ coding sequence is written in lower-case letters.

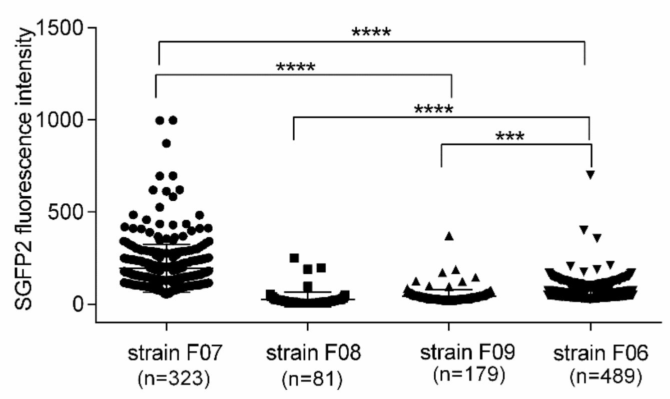

Fig S1. The SGFP2 fluorescence intensities of individual dormant spores of *B. cereus* with wide-field microscopic imaging. The SGFP2 fluorecence intensities of strains F07 (GerRA-SGFP2 and GerD-mScarlet-I), F08 (GerRC-SGFP2 and GerD-mScarlet-I), F09 (GerRB-SGFP2 and GerD-mScarlet-I), and F06 (GerR(A-C-B)-SGFP2 and GerD-mScarlet-I) were measured by the ObjectJ plungin SporeAnalyzer_1c.ojj. ***: $p < 0.001$ and ****: $p < 0.0001$.
